# Commentary: Learning points from a remarkable success in achieving stable pulmonary blood flow in a very small baby with Ebstein anomaly and a circular shunt

**DOI:** 10.1016/j.xjtc.2021.02.028

**Published:** 2021-02-26

**Authors:** Gananjay G. Salve, David S. Winlaw

**Affiliations:** a Heart Centre for Children, The Children's Hospital at Westmead, Westmead, Australia; b The Heart Institute, Cardiothoracic Surgery, Cincinnati Children's Hospital Medical Center, Cincinnati, Ohio

**Figure:**
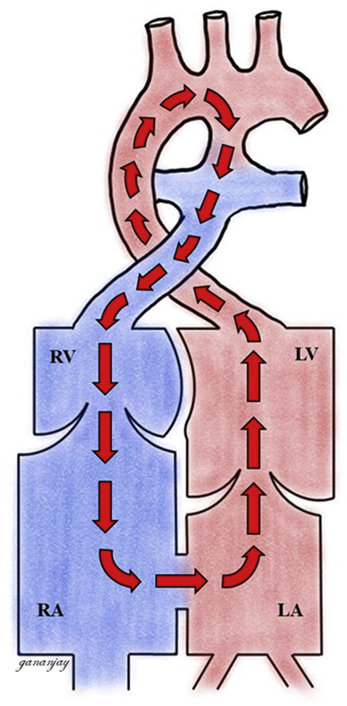
Sketch diagram showing the circular shunt in severe Ebstein anomaly.

**Central Message**

Securing stable pulmonary blood flow in a low-birth-weight baby after a Starnes procedure is difficult. Closing the main pulmonary artery and banding the pulmonary arteries is a good interim stage.

See Article page 208.

Critical neonatal Ebstein anomaly with persistent circular shunt is a difficult and complex problem, with a perinatal mortality as high as 45%.^1^ It is uncommon and frequently fatal. According to the Society of Thoracic Surgeons Congenital Heart Surgery Database (2015-2019), Ebstein anomaly constituted only 0.5% of all primary neonatal diagnoses,^2^ revealing its rare occurrence. It is difficult to know when to intervene: a Starnes procedure addresses the problematic physiology, but the management of the often sick, preterm, and very low birth weight baby after a bypass operation is further complicated by the myriad complexities of managing the systemic to pulmonary shunt.

Deng and colleagues^3^ did a remarkable job of rescuing a premature, very low birth weight neonate with Ebstein anomaly and persistent circular shunt. They performed a “rapid staged repair,” which finally involved retention of the patent ductus arteriosus (PDA) and bilateral branch pulmonary artery (PA) bands to have a controlled pulmonary circulation instead of performing a modified Blalock–Taussig–Thomas (BTT) shunt, which commonly accompanies the modified Starnes procedure. This rescued the patient with a short period of extracorporeal membrane oxygenation. A modified BTT shunt was eventually done 3 months later along with takedown of PA bands and PDA ligation.

The authors used this approach after initial PA ligation and right atrial reduction failed to stabilize the baby. It would be reasonable to move straight to main PA ligation and PA banding along with right ventricular exclusion in future cases. It is easy to make this suggestion in retrospect, and we acknowledge this group's experience in this condition. Nevertheless, in management of Ebstein anomaly, sometimes an early and substantial intervention is the best way to correct an otherwise downward trajectory, particularly when a circular shunt exists. This needs to be balanced against the well-justified approach of medical management only—improving forward flow by lowering pulmonary resistance, improving right ventricular function, and allowing progressive ductal closure. Perhaps the message here is, if something is required to be done, be sure to make it as definitive as possible.

There have been a number of reports using this approach in severe neonatal Ebstein anomaly with circular shunt.^4^ One of the reported patients required a PDA stent and survived through the subsequent stages, whereas the other died. Another case report mentions use of bilateral branch PA banding with PDA for a short period of 4 days, after which it was converted to a Starnes procedure with a modified BTT shunt.^5^ The variability in outcomes highlights the fragility of these neonates, the difficulties involved in achieving a stable circulation, and the fact that PA banding with an open duct is not a panacea, making the reported outcome all the more noteworthy.
